# Racial and Ethnic Disparities in Perceived Healthcare Discrimination and Health Outcomes

**DOI:** 10.1007/s11606-025-09627-y

**Published:** 2025-05-30

**Authors:** Daniel Sabater Minarim, Kylie M. Morgan, Kieran Buckley, Paul Riviere, Carol Ochoa, Leah N. Deshler, Elizabeth A. Duran, Winta T. Mehtsun, Brent S. Rose, Matthew P. Banegas

**Affiliations:** 1 Center for Health Equity Education and Research, University of California, La Jolla, CA USA; 2 Department of Radiation Medicine and Applied Sciences, University of California San Diego School of Medicine, La Jolla, CA USA; 3 School of Biological Sciences, University of California, San Diego, La Jolla, CA USA; 4 Division of Surgical Oncology, University of California San Diego School of Medicine, La Jolla, CA USA

**Keywords:** healthcare discrimination, overall health, health disparities

## Abstract

**Background:**

Experiencing discrimination in the healthcare system may have important implications for health outcomes among racial and ethnic minoritized groups and is a key factor in the disparities observed in healthcare across different races and ethnicities. This study’s objective is to examine the relationship between perceived healthcare discrimination and self-reported health status among racial and ethnic minorities.

**Methods:**

This is a cross-sectional analysis of data from the “All of Us” research program. The study uses the Discrimination in Medical Settings Scale and Overall Health Survey to assess healthcare discrimination and health status. Multivariable logistic regression models were used, adjusting for demographic and socioeconomic characteristics. Adults 18 years or older with self-identified racial and ethnic backgrounds who completed surveys on healthcare discrimination and health status were included. Participants identified as Asian, Black or African American (AA), Hispanic or Latino, non-Hispanic White (NHW), Multiracial, and Other.

**Results:**

The study analyzed data from 92,321 participants, among whom 78% were NHW, Hispanic/Latinos (8%), Black/AAs (7%), and others (7%). Approximately 49% (3070) of Black/AA, 39% (2839) Hispanic/Latinos, and 49% (768) of Multiracial individuals reported experiencing healthcare discrimination, along with 35% (25338) of Whites. Participants who experienced healthcare discrimination had higher odds of self-reported poor health status (aOR 2.19, 95%CI 2.12–2.26). These associations persisted across all analyzed racial and ethnic groups in adjusted models.

**Discussion:**

This study highlights the association between perceived healthcare discrimination and poor self-reported health among racial and ethnic minoritized individuals. Implementing strategies to reduce discrimination within the healthcare system, such as cultural humility training for all staff and inclusive healthcare policies, is critical for improving healthcare quality and health equity.

**Graphical Abstract:**

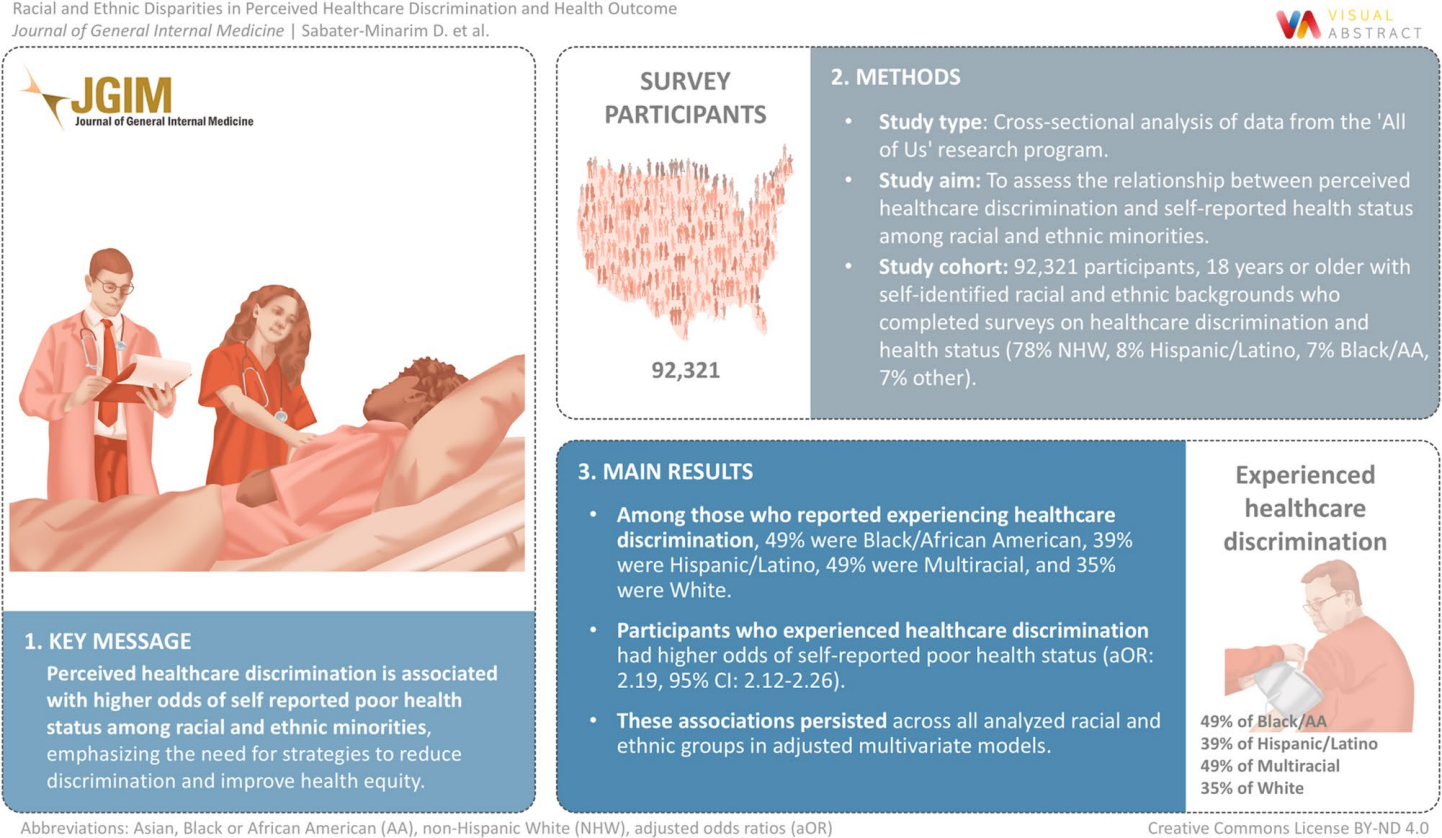

**Supplementary Information:**

The online version contains supplementary material available at 10.1007/s11606-025-09627-y.

## INTRODUCTION

Health disparities among under-represented racial and ethnic groups are deeply rooted in interpersonal, institutional, and systemic biases in policies and practices within the healthcare system. These structural inequities are drivers of adverse health outcomes, serving as barriers to healthcare services.^1–3^ There has been extensive research that shows racial and ethnic minoritized populations experiencing some of the highest rates of chronic diseases, lower life expectancy, and poorer health outcomes compared to their White counterparts.^4^ These differences are due, in large part, to long-standing systemic inequities.^5,6^ Healthcare discrimination can create a preconceived notion that significantly influences patients’ view of the healthcare system’s role in their health. Recent evidence highlights that over one-third of adults reported experiencing at least one form of discrimination in healthcare settings, with higher prevalence among non-Hispanic Black individuals and those with disabilities.^7^ Some historical injustices like the United States Public Health Untreated Syphilis Study and the exploitation of the Havasupai Tribe in genetic research are just a few of the many institutionalized injustices that have fostered mistrust and, therefore, underutilization of the healthcare system by minoritized individuals.^8,9^

Discrimination can manifest through various channels, including unequal treatment by healthcare providers, inadequate access to medical resources, and biased medical advice.^10^ For example, discrimination can impact an individual’s willingness to seek necessary care, which can have a compounding effect on untreated chronic conditions or receiving necessary preventative care.^11^ Further, individuals who have experienced this discrimination will be deterred from seeking care, stopping treatment early, or finding treatment helpful at all.^12^ These practices can exacerbate existing health disparities, such as those observed during the COVID-19 epidemic, during which many historically under-represented and under-served populations had low rates of vaccination, high rates of COVID-19 infection, and mortality. Such intersectionality between socioeconomic status, race, ethnicity, sex, and gender identity compounds these health inequities.^13,14^ We also conceptualize intersectionality as rooted in broader systems of power and oppression, building on the foundational work by Crenshaw.^15^ This lens illuminates how multiple and overlapping social identities can expose individuals to layers of discrimination.

Previous studies have investigated the association between perceived healthcare discrimination and health outcomes in a limited scope, with smaller populations and regions looking at specific forms of discrimination. These studies have also not explored the intersectionality of various factors, such as socioeconomic status, race, ethnicity, gender identity, sexual orientation, and their influence on perceived healthcare experiences and health.^16,17^ Our study will fill this gap in literature by utilizing one of the largest and most diverse prospective cohorts in the USA. We will consider the intersectionality of various demographic and socioeconomic factors that will provide new insights into the impact of healthcare discrimination on health outcomes, comprehensively contributing to existing literature.

## METHODS

### Data Source and Research Platform

Our cross-sectional analysis was conducted using the “All of Us” research program, which aims to enroll a diverse group of at least 1 million individuals in the USA to help accelerate biomedical research and improve health outcomes.^18^ Since May 2018, this program has been collecting extensive biomedical information, including patient demographics, electronic health records (EHRs), health questionnaires, social determinants of health surveys, physical measurements, digital health technology, and biospecimen analysis.^19,20^ The program protocol follows the Declaration of Helsinki and is approved by the National Institutes of Health Institutional Review Board. Informed consent is received from all participants, and their data is coded to be de-identified.

### Participants

We included participants 18 years or older who completed the healthcare discrimination survey and responded to self-rated health status questions; this encompassed survey data from November 2020 to June 2022. The demographic information included age at the time of the survey, self-reported sex (female, male, or various gender-diverse options), self-reported sexual orientation (heterosexual and diverse sexual orientation), and race/ethnicity. For race/ethnicity, participants self-identified as either Asian, Black or African American (AA), Multiracial, non-Hispanic White (NHW), or Other. Individuals identifying as Hispanic or Latino were classified under this ethnicity regardless of their original racial categorization. We also included marital status, nativity of USA or foreign born, annual income grouped in < 35 K to >150 K, level of education (advanced degree, high school degree, less than high school), health insurance, and whether English is their second language. Disability status was determined based on a series of ADA-compliant questions asking whether the respondent had difficulties in hearing, seeing, concentrating, walking, dressing, bathing, or running errands.

### Exposure — Perceived Healthcare Discrimination

Healthcare discrimination was assessed using the Discrimination in Medical Settings Scale (DMS) from the “All of Us” Social Determinants of Health survey.^21^ This scale included seven questions asking participants how often they experienced these discriminatory behaviors when visiting a doctor’s office or other healthcare providers. These questions included being treated with less courtesy or respect than other people, receiving poorer service than others, having a doctor or nurse act as if they thought the participant was not smart, having a doctor or nurse act as if they were afraid of the participant, having a doctor or nurse act as if they were better than the participant, and feeling like a doctor or nurse was not listening to what the participant was saying.

Participants could respond to these questions with “Never,” “Rarely,” “Sometimes,” “Most of the time,” and “Always.” For all seven measures, those who responded “Sometimes,” “Most of the time,” or “Always” were defined as having experienced healthcare discrimination. We created a binary, combined “Overall Discrimination” measure, in which participants who experienced discrimination in ≥1 or more of the seven items were defined as “experienced any healthcare discrimination,” and all those who did not experience discrimination were defined as “did not experience any healthcare discrimination.”^22^

### Outcome — Self-reported Health Status

Self-reported health status was gauged using the Participant Provided Information (PPI) survey. These questions asked participants how they rate their physical health, mental health, and, in general, their overall health.

Responses were on a 5-point scale ranging from “Poor” (1) to “Excellent” (5). Participants who reported their physical, mental, or general health as “Fair” or “Poor” were categorized as having “poor health.”

### Statistical Analysis

Descriptive statistics were calculated for participant demographic, socio-economic, discrimination, and health status characteristics. Analysis of variance and Chi-square test were conducted to test the differences of mean age and categorical variables across racial/ethnic groups. For each healthcare discrimination survey question and the aggregated overall discrimination variable, we used multivariable logistical regression to find the association between perceived healthcare discrimination and race, controlling for demographic and socioeconomic factors. Multivariable logistic regression models were used to explore the association between perceived healthcare discrimination and self-reported poor health, after controlling for demographic and socioeconomic factors. In addition, we incorporated two-way interaction terms (race × gender, race × disability, and race × sexual orientation) in separate logistic regression models to examine potential intersectional effects on perceived discrimination. We also reported point estimates as odds ratios (ORs) and their 95% confidence intervals (CIs). All the statistical tests were two-sided, at the significance level *p* < 0.05. All the analysis was performed with the Researcher Workbench, using a cloud-based platform for a Jupyter Notebook accessed through the “All of Us” program, powered by Python version 3.8.1.

## RESULTS

### Demographic Characteristics

Table 1 presents the characteristics of the 92,321 participants, who predominantly consisted of NHW participants (78%), followed by Hispanic/Latino (8%), Black/AA (7%), Asian (3%), Multiracial (2%), and Others (2%). The mean age varied from 45 years among Asians and Multiracial individuals to 59 years for Others.

**Table 1:** Study Sample Characteristics

Variable		Asian	Black/African American	Hispanic/Latino	Multi-race	Other	White
	Total *n*=92,321	*n*=2538 (3%)	*n*=6265 (7%)	*n*=7279 (8%)	*n*=1568 (2%)	*n*=2275 (2%)	*n*=72,396 (78%)
Mean age (S.D.)		45 (17)	53 (14)	46 (15)	45 (16)	59 (16)	57 (16)
Nativity	US born	1062 (42%)	5888 (94%)	4459 (61%)	1376 (88%)	1851 (81%)	69,456 (96%)
	Foreign born	1476 (58%)	377 (6%)	2820 (39%)	192 (12%)	424 (19%)	2940 (4%)
Linguistics	English first language	1498 (59%)	6121 (98%)	4843 (67%)	1518 (97%)	1980 (87%)	71,468 (99%)
	English second language	1040 (41%)	144 (2%)	2436 (33%)	50 (3%)	295 (13%)	928 (1%)
Marital Status	Married	1326 (52%)	1899 (30%)	3378 (46%)	731 (47%)	1263 (56%)	44,240 (61%)
	Not married	1212 (48%)	4366 (70%)	3901 (54%)	837 (53%)	1012 (44%)	28,156 (39%)
Gender	Male	982 (39%)	1604 (26%)	2139 (29%)	436 (28%)	903 (40%)	26,206 (36%)
	Female	1503 (59%)	4588 (73%)	5000 (69%)	1077 (69%)	1314 (58%)	44,886 (62%)
	Diverse genders	53 (2%)	73 (1%)	140 (2%)	55 (4%)	58 (3%)	1304 (2%)
Sexual orientation	Straight	2214 (87%)	5643 (90%)	6150 (84%)	1212 (77%)	1984 (87%)	63,933 (88%)
	Non-heterosexual	324 (13%)	622 (10%)	1129 (16%)	356 (23%)	291 (13%)	8463 (12%)
Insurance status (*N*%)	Insured	2464 (97%)	5824 (93%)	6611 (91%)	1533 (98%)	2207 (97%)	71182 (98%)
	Uninsured	74 (3%)	441 (7%)	668 (9%)	35 (2%)	68 (3%)	1214 (2%)
Income	Less than 35k	407 (16%)	2983 (48%)	2807 (39%)	345 (22%)	611 (27%)	11,731 (16%)
	35k–75k	490 (19%)	1702 (27%)	2012 (28%)	389 (25%)	606 (27%)	18,702 (26%)
	75k–150k	816 (32%)	1141 (18%)	1597 (22%)	481 (31%)	625 (27%)	25,275 (35%)
	More than 150k	825 (33%)	439 (7%)	863 (12%)	353 (23%)	433 (19%)	16,688 (23%)
Education	Advanced degree	2175 (86%)	2756 (44%)	3524 (48%)	1090 (70%)	1461 (64%)	50,340 (70%)
	High school	353 (14%)	3161 (50%)	3032 (42%)	459 (29%)	755 (33%)	21,330 (29%)
	Less than high school	<20 (0%)	348 (6%)	723 (10%)	<20 (1%)	59 (3%)	726 (1%)
Disability	No	2379 (94%)	5186 (83%)	6240 (86%)	1323 (84%)	1846 (81%)	63,560 (88%)
	Yes	159 (6%)	1079 (17%)	1039 (14%)	245 (16%)	429 (19%)	8836 (12%)

Marital status: Married includes those who are partnered and or living with partner

Insurance status: Includes all public, government, and private insurance

Income: household income

Disability status: determined based on a series of ADA-compliant questions asking if respondent had difficulties in hearing, seeing, concentrating, walking, dressing, bathing, or running errands

Our study had predominantly US-born Black or AA (94%) and NHW (96%) participants compared to a substantial number of foreign-born Asian (58%) and Hispanic/Latino (39%) participants. With respect to sexual orientation, a significant portion in each group identifies as non-heterosexual (10–23%), with the highest in the Multiracial group. It is also worth noting the amount of insurance coverage; it is very high in all groups and ranges from 91 to 98% of participants. There is an income discrepancy: 48% of Black/AA and 39% of Hispanics/Latinos, respectively, earn less than $35,000, while only 7% of NHW have this income level. The differences across all the demographic and social variables are statistically significant in Table 1.

### Prevalence of Healthcare Discrimination

**Figure 1:**
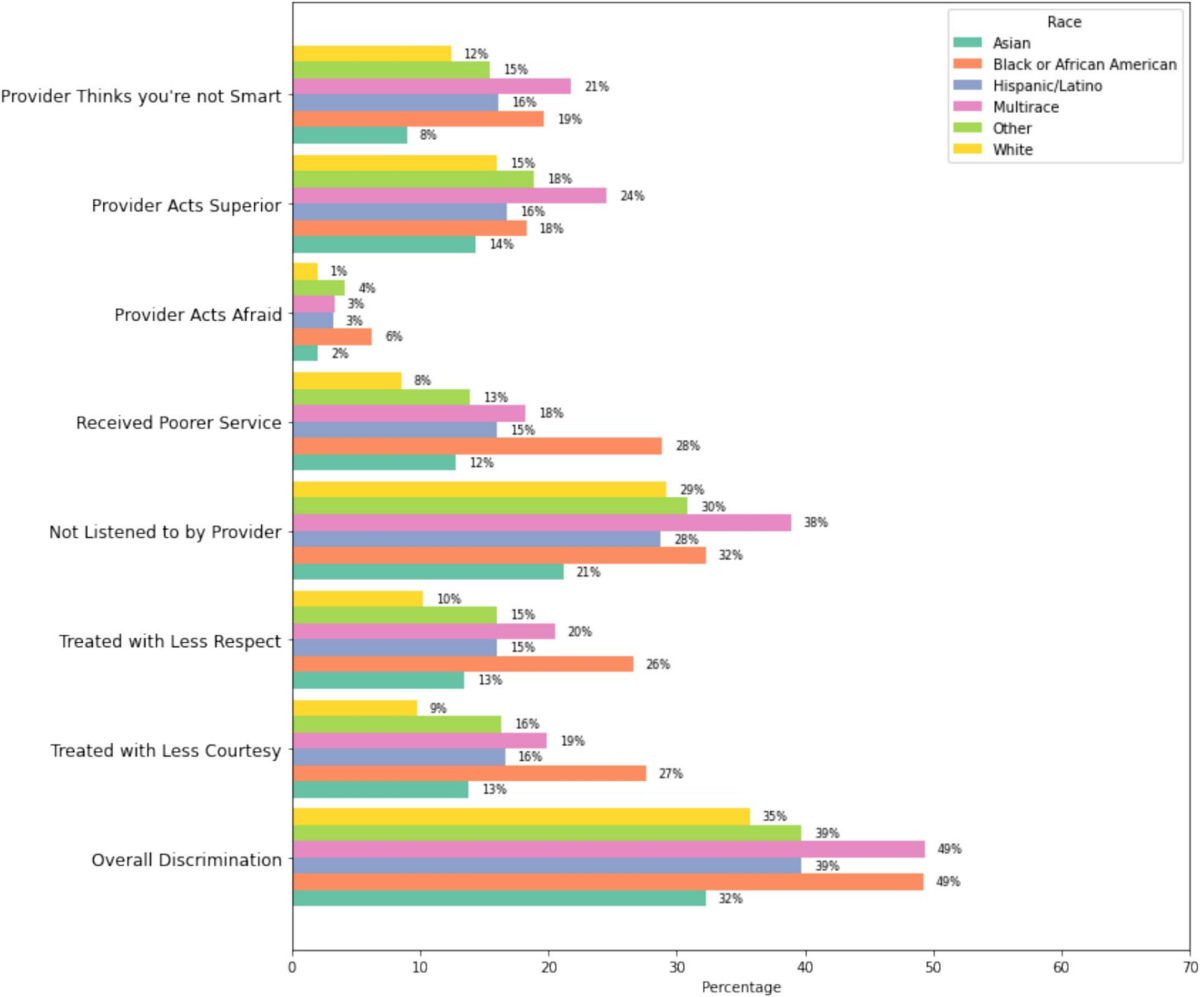
Percentage of self-reported health care discrimination by race and ethnicity.

Figure 1 delineates participants’ responses to the healthcare discrimination items. Among Black or AA participants, a greater proportion reported being treated with less courtesy (27%), being treated with less respect (26%), receiving poorer service (28%), and feeling that the provider acts afraid of them (6%) than other racial/ethnic groups. Multiracial participants had a greater proportion who reported feeling that the provider thought they were not smart (21%), the provider acted superior (24%), and not being listened to by the provider (49%) than other groups. NHW and Asian participants had the lowest percentage of individuals reporting healthcare discrimination across all measures. Asian individuals report feeling disrespected at 13% and not listened to at 21%. Participants categorized as Other experience being treated as inferior at 18% and not being listened to at 39%. Across all participants, feeling not listened to emerges as a common form of discrimination, while the provider acting afraid was the least commonly experienced type of discrimination.

### Healthcare Discrimination by Race/Ethnicity

Table 2 adjusted analyses reveal significant disparities in experiences of healthcare discrimination. Black or AA participants demonstrated higher odds of discrimination across most measures compared to NHW participants. Notably, they experienced higher odds of being treated with less courtesy (aOR 2.69, 95%CI 2.52–2.87), less respect (aOR 2.40, 95%CI 2.25–2.56), receiving poorer service (aOR 3.53, 95%CI 3.31–3.76), and providers acting as if they are afraid (aOR 2.84, 95%CI 2.51–3.20).

**Table 2:** Adjusted Odds Ratios of Experiencing Healthcare Discrimination

Health Care Discrimination Survey question	Asian	Black or African- American	Hispanic/Latino	Multi-race	Other
How often are you treated with less courtesy than other people when you go to a doctor's office or other health care provider?	1.35 (1.19−1.54)	2.69 (2.52−2.87)	1.23 (1.14−1.33)	1.67 (1.46−1.90)	1.71 (1.52−1.93)
How often are you treated with less respect than other people when you go to a doctor's office or other health care provider?	1.23 (1.09−1.40)	2.40 (2.25−2.56)	1.10 (1.02−1.19)	1.59 (1.39−1.81)	1.59 (1.41−1.79)
How often do you feel like a doctor or nurse is not listening to what you were saying when you go to a doctor's office or other health care provider?	0.59 (0.54−0.66)	0.93 (0.88−0.99)	0.77 (0.73−0.82)	1.08 (0.97−1.2)	1.12 (1.02−1.24)
How often do you receive poorer service than others when you go to a doctor's office or other health care provider?	1.44 (1.26−1.64)	3.53 (3.31−3.76)	1.46 (1.35−1.58)	1.76 (1.55−2.03)	1.65 (1.45−1.87)
How often does a doctor or nurse act as if he or she is afraid of you when you go to a doctor's office or other health care provider?	0.95 (0.70−1.27)	2.84 (2.51−3.20)	1.29 (1.10−1.52)	1.48 (1.11−1.96)	1.93 (1.57−2.40)
How often does a doctor or nurse act as if he or she is better than you when you go to a doctor's office or other health care provider?	0.77 (0.68−0.87)	0.97 (0.90−1.04)	0.81 (0.75−0.87)	1.17 (1.04−1.32)	1.26 (1.13−1.40)
How often does a doctor or nurse act as if he or she thinks you are not smart when you go to a doctor's office or other health care provider?	0.60 (0.52−0.70)	1.37 (1.28−1.47)	0.98 (0.91−1.06)	1.34 (1.18−1.52)	1.29 (1.14−1.46)
Overall discrimination	0.78 (0.71−0.86)	1.40 (1.33−1.48)	0.89 (0.84−0.95)	1.27 (1.14−1.41)	1.21 (1.10−1.31)

Covariates included in regression: insurance, marriage, English second language, sexual orientation, gender, disability, education, birthplace using non-Hispanic White as the reference group

Marital status: Married includes those who are partnered and or living with partner

Insurance status: Includes all public, government and private insurance

Income: household income

Disability status: determined based on a series of ADA-compliant questions asking if respondent had difficulties in hearing, seeing, concentrating, walking, dressing, bathing, or running errands

Multiracial participants reported higher odds of discrimination, particularly being treated with less courtesy (aOR 1.67, 95%CI 1.46–1.90), less respect (aOR 1.59, 95%CI 1.39–1.81), and receiving poorer service (aOR 1.76, 95%CI 1.55–2.03). Additional notable findings include perceptions of provider superiority (aOR 1.17, 95%CI 1.04–1.32) and assumptions of lower intelligence (aOR 1.34, 95%CI 1.18–1.52).

Hispanic or Latino participants also faced discrimination in being treated with less courtesy (aOR 1.23, 95%CI 1.14–1.33) and receiving poorer service (aOR 1.46, 95%CI 1.35–1.58).

Beyond racial and ethnic differences, diverse gender individuals had significantly higher odds of experiencing healthcare discrimination (aOR 2.39, 95%CI 2.14–2.66), and non-heterosexual individuals (aOR 1.49, 95%CI 1.33–1.69) (Supplementary Table 2).

### Association Between Healthcare Discrimination and Poor Health

As shown in Table 3, individuals who experienced healthcare discrimination reported significantly higher odds of self-reported poor overall health (aOR 2.19, 95%CI 2.12–2.26). Black or African American participants had higher odds of poor health (aOR 1.21, 95%CI 1.14–1.29), followed closely by Hispanic/Latino (aOR 1.16, 95%CI 1.09–1.24) and Other racial groups (aOR 1.16, 95%CI 1.05–1.29), compared to NHW. Other factors found to be associated with self-reported poor overall health included being non-heterosexual (aOR 1.49, 95%CI 1.33–1.69), disabled (aOR 4.28, 95%CI 1.33–1.69), and not married (aOR 1.44, 95%CI 1.44). These patterns remained in race/ethnicity-stratified models.

**Table 3:** Association Between Healthcare Discrimination and Self-reported Poor Health

Variable	Value	OR (95% CI)	*p*-value
Experienced healthcare discrimination (ref: No)	Yes	2.19 (2.12− 2.26)	<0.01
Race (ref: NHW)	Asian	1.0 (0.90− 1.11)	0.99
	Black or African-American	1.21 (1.14− 1.29)	< 0.01
	Hispanic/Latino	1.16 (1.09− 1.24)	< 0.01
	Multi-race	0.95 (0.84− 1.07)	0.37
	Other	1.16 (1.05− 1.29)	< 0.01
Education (ref : less than high school)	Per unit increase (high school- advanced degree)	0.48 (0.46− 0.50)	< 0.01
Age	Per 1-year increase	0.98 (0.98− 0.98)	< 0.01
Birthplace (ref: US born)	Foreign born	0.91 (0.86− 0.98)	< 0.01
Insurance status (ref: Insured)	Uninsured	1.08 (0.99− 1.19)	0.08
Gender (ref: Male)	Diverse genders	1.49 (1.33− 1.69)	< 0.01
	Female	1.03 (1.0− 1.07)	0.09
Linguistics (ref: English first language)	English second language	0.93 (0.84− 1.02)	0.07
Disability status (ref: no disability)	Disability	4.28 (4.09− 4.47)	< 0.01
Marital status (ref: married)	Not married	1.44 (1.40− 1.49)	< 0.01
Sexual orientation (ref: heterosexual)	Non-heterosexual	1.38 (1.32− 1.45)	< 0.01

Marital status: Married includes those who are partnered and/or living with a partner

Insurance status: Includes all public, government, and private insurance

Income: household income

Disability status: determined based on a series of ADA-compliant questions asking if respondent had difficulties in hearing, seeing, concentrating, walking, dressing, bathing, or running errands

## DISCUSSION

Our study identifies the experiences of healthcare discrimination within healthcare systems on health outcomes, particularly among individuals from racial and ethnic minoritized groups. Black or AA participants reported the highest levels of perceived healthcare discrimination, including being treated with less courtesy and less respect, encountering providers acting afraid, and receiving poorer service. Asian participants reported lower rates of discrimination, yet notable disparities were still observed compared to the non-Hispanic White reference group. Additionally, individuals who selected Other reported experiences of discrimination, such as being treated as inferior and not being listened to by providers. Meanwhile, Multiracial participants reported distinctly high levels of discrimination from providers acting superior, smarter, and concerns not being listened to. This aspect is particularly concerning given the rapid growth of the Multiracial population. We also found that experiencing healthcare discrimination is associated with significantly higher odds of self-reported poor overall health than those individuals who do not experience such discrimination. Similar findings have been reported in cancer-specific populations, where perceived discrimination was linked to worse health-related quality of life.^23^ Consequently, this research helps quantify the extent to which healthcare discrimination is experienced across diverse population groups and the different ways in which discrimination is manifested, and, importantly, indicates a clear association with patient-reported health outcomes.

There were higher rates of healthcare discrimination among Black/AA and Multiracial individuals, compared to existing research. For instance, a study by Bleich et al. (*n*=902) reported 32% of non-Hispanic Black respondents experienced discrimination in healthcare settings, which is lower compared to the 49% (*n*=92,000) reported in our sample.^24^ Similarly, Multiracial individuals in our study reported high rates of discrimination, a finding challenging to compare since they are underrepresented in existing research. Moreover, the study by Nong et al. (*n*=2137) found that 22.9% of Hispanic respondents reported discrimination. In contrast, our study reported a higher figure of 39% among Hispanic/Latino participants.^25^ This difference could be partly attributed to our larger sample size and our use of a multi-item discrimination in medical settings scale, capturing a more accurate prevalence of discrimination. Additionally, subdividing the Hispanic category into sub-groups such as Hispanic White, Hispanic Black, and others might reveal even more distinct patterns of discrimination. These distinctions elucidate variations in discrimination experiences within the Hispanic community. The study by Abramson et al., conducted solely in California, found that African Americans are 3.6 times more likely to report discrimination compared to Whites. While this study investigated geographic-specific disparities, our results reflect significant trends in racial disparities and provide a valuable foundation for understanding healthcare experiences at a broader level.^26^ Asian participants reported relatively lower levels of discrimination; however, they still experienced significant disparities such as receiving poorer service (aOR 1.44) and being treated with less respect (aOR 1.23). As shown in the study by Gee et al., subtle forms of discrimination, such as being treated with less respect, lie beneath overt bias and significantly impact patient health outcomes, emphasizing the need to address these inequities to reduce health disparities.^27^ Individuals who selected Other racial/ethnic groups reported significant discrimination, such as providers acting afraid (aOR 1.93) and being treated with less courtesy (aOR 1.71).This aligns with a review by Hamed et al., which highlighted that those not fitting predefined racial/ethnic categories often face unique forms of discrimination, resulting in adverse health outcomes.^28^

Intersectionality is crucial in understanding the complexities of healthcare discrimination.^29^ Specifically, individuals with multiple identities (i.e., belonging to more than one “group”) may uniquely experience various forms of discrimination that align with their converging identities. Our study points to various disparities in discrimination experiences among individuals with distinct identities (Supplementary Table 2) — such as diverse-gender individuals, those with disabilities, and non-heterosexual individuals — reporting higher odds of discrimination compared to their counterparts (aORs 2.39, 1.87, and 1.48, respectively). Yet, in our intersectional analyses (Supplementary Table 3), it reveals sub-additive or non-significant interactions, although other research has documented more pronounced or additive effects in different contexts. This underscores the multifaceted nature of discrimination and highlights the importance of continuing to explore how intersecting identities shape health experiences.^30,31^ These findings emphasize the need to examine how intersecting systems of power, such as racism, sexism, ableism, and heteronormativity, shape healthcare experiences rather than solely focusing on individual identities. By connecting these systems to the observed disparities, our analysis demonstrates how structural inequities influence healthcare experiences.

Education also played a significant role in the experience of healthcare discrimination and overall health outcomes. In Supplementary Table 2, higher education was inversely related to the likelihood of encountering healthcare discrimination (aOR 0.82, 95%CI 0.8–0.84). Furthermore, Table 2reveals that each increase in education level (from high school to advanced degree) had significantly lower odds of reporting poor overall health (OR 0.48, 95%CI 0.46–0.50). Highlighting the protective effect of higher education against poor health outcomes and how literacy and comprehension of what can be complex issues such as one’s health can help one’s well-being.^31^

To address the current study’s limitations, further research should investigate the broader effects of healthcare discrimination. This includes conducting a longitudinal study to better understand the long-term effects of discrimination on health outcomes and care adherence. Expanding the study to a wider array of racial and ethnic groups to help understand the differences in perceived healthcare discrimination. Being able to understand these differences and the specific mechanism that discrimination undermines healthcare will help enhance access to services and quality of care.

### Limitations

Our cross-sectional non-weighted study limits the ability to draw inferences between perceived healthcare discrimination and health outcomes at a national level. Also, relying on self-reported variables can create the possibility of response bias. Participants’ perceptions of discrimination can also be influenced by their subjective experiences or societal narratives, which could overstate or understate the incidence of discrimination in healthcare settings. These perceptions are extremely relevant when considering historical and ongoing experiences of racism and discrimination faced by minority groups. Such biases could impact the interpretation of our findings, requiring cautious extrapolation of the results to broader populations. Therefore, our study could benefit from including more representation across all racial and ethnic groups. In future studies, considering the classification of race and ethnicity, to properly capture the complexity of these groups, such as Black Hispanic/Latino to White Hispanic/Latino. The lack of representation of certain racial and ethnic groups, such as Middle Eastern, American Indian, Alaska Native, and Pacific Islanders, also limits the scope of the study. Furthermore, language barriers may further confound the relationship between healthcare discrimination and perceived health, which are not included in this study.

## CONCLUSION

In conclusion, our study highlights the increased prevalence of healthcare discrimination, specifically for racial/ethnic minorities, and its effect on overall health. These disparities can be minimized with systemic and legislative change from the healthcare system, policymakers, and large-scale providers via training. This includes implementing cultural humility training, policies to enhance access to high-quality care for marginalized communities, and active engagement of these communities and stakeholders in healthcare planning. In addition, we can prioritize research on the intersectionality of race, ethnicity, and health while committing to data transparency to ensure biases are mitigated. The need to address healthcare discrimination and its impact on health disparities is a matter of social justice and will be critical to improving public health.

## Supplementary Information

Below is the link to the electronic supplementary material.

Supplementary file1 (DOCX 53 KB)

## Data Availability

The data supporting the findings of this study are available through the All of Us Research Program. Access requires approval and a registered research account.
