# Supplementary material for: Racial and Ethnic Disparities in Perceived Healthcare Discrimination and Health Outcomes
Source: J Gen Intern Med. 2025 May 30;40(11):2548–57. doi: 10.1007/s11606-025-09627-y (PMC12405135; doi:10.1007/s11606-025-09627-y)

**Supplementary Information**

**Supplementary:**

**Supplementary Table 1**: Unadjusted Odds Ratios of Experiencing Healthcare Discrimination by Race

|  | **Asian** | **Black or African- American** | **Hispanic/**  **Latino** | **Multi-race** | **Other** |
| --- | --- | --- | --- | --- | --- |
| **Health Care Discrimination Question** | **OR (95% CI)** | **OR (95% CI)** | **OR (95% CI)** | **OR (95% CI)** | **OR (95% CI)** |
| How often are you treated with less courtesy than other people when you go to a doctor's office or other health care provider? | 1.58  (1.40-1.78) | 3.73   (3.51-3.97) | 1.94   (1.81-27) | 2.47  (2.17-2.81) | 1.83   (1.63-27) |
| How often are you treated with less respect than other people when you go to a doctor\'s office or other health care provider? | 1.41   (1.25-1.59) | 3.29   (39-3.50) | 1.73   (1.61-1.85) | 2.37   (2.09-2.70) | 1.68   (1.49-1.89) |
| How often do you feel like a doctor or nurse is not listening to what you were saying when you go to a doctor's office or other health care provider? | 0.66   (0.60-0.73) | 1.07   (1.01-1.14) | 0.94  (0.89-10) | 1.52   (1.37-1.68) | 13   (0.94-1.13) |
| How often do you receive poorer service than others when you go to a doctor's office or other health care provider? | 1.69   (1.49-1.91) | 4.68  (4.40-4.98) | 2.18   (2.03-2.33) | 2.53   (2.21-2.90) | 1.75   (1.54-1.99) |
| How often does a doctor or nurse act as if he or she is afraid of you when you go to a doctor's office or other health care provider? | 1.33   (0.94-1.87) | 5.57   (4.88-6.35) | 2.46  (29-2.90) | 2.23  (1.58-3.14) | 2.96  (2.30-3.82) |
| How often does a doctor or nurse act as if he or she is better than you when you go to a doctor's office or other health care provider? | 0.87   (0.77-0.98) | 1.13   (1.05-1.21) | 1.03  (0.96-1.10) | 1.68  (1.49-1.89) | 1.19  (16-1.32) |
| How often does a doctor or nurse act as if he or she thinks you are not smart when you go to a doctor's office or other health care provider? | 0.72   (0.63-0.83) | 1.76  (1.64-1.88) | 1.38  (1.29-1.48) | 2.01  (1.77-2.27) | 1.27   (1.12-1.43) |
| Overall Discriminated | 0.87   (0.80-0.95) | 1.73   (1.64-1.82) | 1.18   (1.12-1.24) | 1.77   (1.60-1.96) | 1.18   (18-1.28) |

*** Using Non-Hispanic White as the Reference Group**

**Supplementary Table 2**. Predictors of Perceived Health Care Discrimination

|  | **OR (95% CI)** | **P-value** |
| --- | --- | --- |
| **Education** | 0.82 (0.8-0.84) | <0.01 |
| **Age** | 0.98 (0.98-0.98) | <0.01 |
| **Asian** (ref: NHW) | 0.77 (0.7-0.85) | <0.01 |
| **Black or African-American** (ref: NHW) | 1.39 (1.32-1.47) | <0.01 |
| **Hispanic/Latino** (ref: NHW) | 0.91 (0.86-0.97) | <0.01 |
| **Multirace** (ref: NHW) | 1.27 (1.14-1.41) | <0.01 |
| **Other Race** (ref: NHW) | 1.20 (1.1-1.31) | <0.01 |
| **Birthplace: Foreign Born**  (ref: US Born) | 0.86 (0.82-0.91) | <0.01 |
| **Uninsured**  (ref: Insured) | 1.21 (1.11-1.32) | <0.01 |
| **Diverse Genders** (ref: Male) | 2.39 (2.14-2.66) | <0.01 |
| **Female** (ref: Male) | 1.69 (1.64-1.74) | <0.01 |
| **English Second Language** (ref: English First Language) | 0.95 (0.86-1.05) | 0.3 |
| **Disability: Yes** (ref: No Disability) | 1.87 (1.79-1.95) | <0.01 |
| **Not Married** (ref: Married) | 1.18 (1.14-1.21) | <0.01 |
| **Non-Heterosexual** (ref: Heterosexual) | 1.48 (1.42-1.55) | <0.01 |

*Marital Status: Married includes those who are partnered and/or living with apartner

*Insurance Status: Includes all public, government, and private insurance

*Income: household income

*Disability Status: determined based on a series of ADA-compliant questions asking if respondent had difficulties in hearing, seeing, concentrating, walking, dressing, bathing, or running errands.

### **Supplementary Table 3.** Adjusted Interaction Effects of Race, Disability, Gender, and Sexual Orientation on Perceived Healthcare Discrimination

| **Interaction Term** | **OR (95% CI)** | **p-value** |
| --- | --- | --- |
| **Race × Disability** |  |  |
| Asian × Disability | 0.948 (0.672–1.337) | 0.761 |
| Black or African American × Disability | 0.740 (0.641–0.855) | <0.01 |
| Hispanic/Latino × Disability | 0.779 (0.674–0.900) | <0.01 |
| Multiracial × Disability | 1.072 (0.792–1.449) | 0.653 |
| Other × Disability | 1.002 (0.799–1.257) | 0.984 |
|  |  |  |
| **Race × Gender** |  |  |
| Asian × Female | 0.815 (0.678–0.980) | 0.029 |
| Asian × Diverse Genders | 0.407 (0.226–0.734) | <0.01 |
| Black or African American × Female | 0.646 (0.572–0.730) | <0.01 |
| Black or African American × Diverse Genders | 0.431 (0.258–0.719) | <0.01 |
| Hispanic/Latino × Female | 0.858 (0.765–0.962) | <0.01 |
| Hispanic/Latino × Diverse Genders | 0.653 (0.446–0.957) | 0.029 |
| Multiracial × Female | 0.925 (0.731–1.172) | 0.520 |
| Multiracial × Diverse Genders | 0.724 (0.377–1.392) | 0.333 |
| Other × Female | 0.802 (0.667–0.965) | 0.019 |
| Other × Diverse Genders | 0.867 (0.473–1.589) | 0.645 |
|  |  |  |
| **Race × Sexual Orientation** |  |  |
| Asian × Non-Heterosexual | 0.606 (0.469–0.784) | <0.01 |
| Black or African American × Non-Heterosexual | 0.891 (0.743–1.067) | 0.209 |
| Hispanic/Latino × Non-Heterosexual | 0.852 (0.740–0.981) | 0.026 |
| Multiracial × Non-Heterosexual | 1.154 (0.889–1.497) | 0.283 |
| Other × Non-Heterosexual | 1.152 (0.883–1.503) | 0.298 |

* Each logistic regression (Race × Disability, Race × Gender, and Race × Sexual Orientation) adjusted for Age, Highest Education, Insurance, Birthplace, English Second Language, Marital Status, plus the respective main effects of Race, Disability, Gender, and Sexual Orientation.

**Supplementary Figure 1**. Adjusted Odds Ratios of Perceived Discrimination in Healthcare Settings by Race


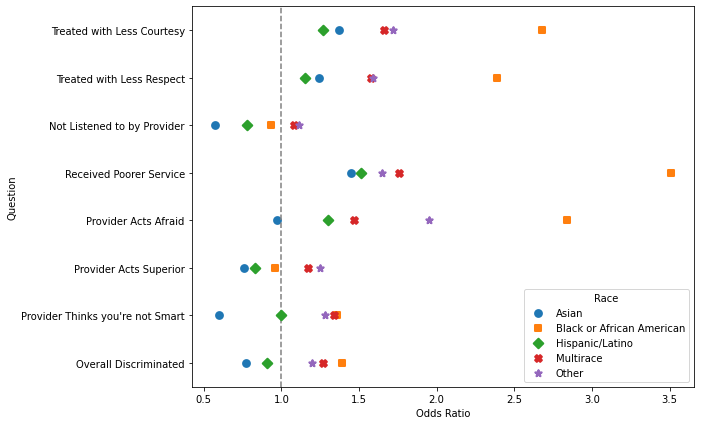

Supplement: Supplementary file 1 — Supplementary file1 (DOCX 53 KB) [file 11606_2025_9627_MOESM1_ESM.docx]
